# Gender disparities in the association between marital status and intention to leave research careers among medical researchers

**DOI:** 10.1265/ehpm.25-00407

**Published:** 2026-05-09

**Authors:** Yuki Kawai, Keisuke Kuwahara, Akira Minoura, Yuhei Shimada, Makoto Kondo, Hiroko Fukushima, Hiroaki Komatsu, Takehiro Sugiyama

**Affiliations:** 1 Department of Epidemiology and Prevention, Center for Clinical Sciences, Japan Institute for Health Security, Tokyo, Japan; 2 Department of Public Health, Yokohama City University School of Medicine, Kanagawa, Japan; 3 Department of Health Data Science, Graduate School of Data Science, Yokohama City University, Kanagawa, Japan; 4 Department of Hygiene, Public Health and Preventive Medicine, Showa Medical University School of Medicine, Tokyo, Japan; 5 Department of Law and Politics, The University of Tokyo, Tokyo, Japan; 6 Diabetes and Metabolism Information Center, National Institute of Global Health and Medicine, Japan Institute for Health Security, Tokyo, Japan; 7 Department of Anatomy and Neuroscience, Graduate School of Medicine, Osaka Metropolitan University, Osaka, Japan; 8 Department of Pediatrics, University of Tsukuba Hospital, Tsukuba, Japan; 9 Department of Child Health, Institute of Medicine, University of Tsukuba, Tsukuba, Japan; 10 Department of Obstetrics and Gynecology, School of Medicine, Tottori University Faculty of Medicine, Tottori, Japan; 11 Department of Health Services Research, Institute of Medicine, University of Tsukuba, Tsukuba, Japan; 12 Department of Medical DX, Bureau of System Infrastructure Development, Japan Institute for Health Security, Tokyo, Japan

**Keywords:** Medical researcher, Gender, Intention to leave, Marital status, Having children

## Abstract

**Background:**

Gender disparities in the intention to leave research careers after marriage have not been investigated. This study aimed to clarify the gender-specific associations of marital status and nurturing children with the intention to leave research careers among medical researchers.

**Methods:**

A cross-sectional study was conducted from December 2022 to January 2023. The study was based on a web-based survey targeting medical researchers affiliated with 141 Japanese academic societies. Participants who disagreed with continuing their research careers were classified as intending to leave. Multivariable-adjusted logistic regression analysis was used to evaluate the gender-specific association of marital status and having children with the intention to leave research careers.

**Results:**

Among 3,009 participants, 812 (27.0%) were women, and 342 (11.4%) expressed an intention to leave. A total of 2,481 participants (82.5%) were married, and 2,267 (75.3%) had children. Marital status was associated with intention to leave research careers in opposite directions for men and women, with significant interactions on both multiplicative (*P* = 0.015) and additive (*P* = 0.001) scales. Specifically, married men had a significantly lower intention to leave compared with unmarried men (adjusted odds ratio [aOR] 0.63, 95% confidence interval [CI]: 0.40–0.97). In contrast, among women, marriage was associated with a higher intention to leave compared with being unmarried (aOR 1.41, 95% CI: 0.86–2.32), although this increase was not significant. In post hoc subgroup analyses by medical license status, the gender-specific association between marital status and intention to leave research careers was more evident among medical doctors (MDs) than non-MDs, among whom the proportion reporting intention to leave was lower (4% to 6% across groups). However, estimates among non-MD researchers were imprecise due to the small number of events. Similar gender disparities were observed for having children, although the interaction did not reach statistical significance (*P* for additive interaction = 0.083).

**Conclusions:**

The present results suggest that marital status showed opposite associations with intention to leave research careers between men and women, a pattern primarily observed among MD researchers. Our finding highlights the need to consider gender difference when developing strategies to support retention of medical researchers.

**Supplementary information:**

The online version contains supplementary material available at https://doi.org/10.1265/ehpm.25-00407.

## Graphical Abstract

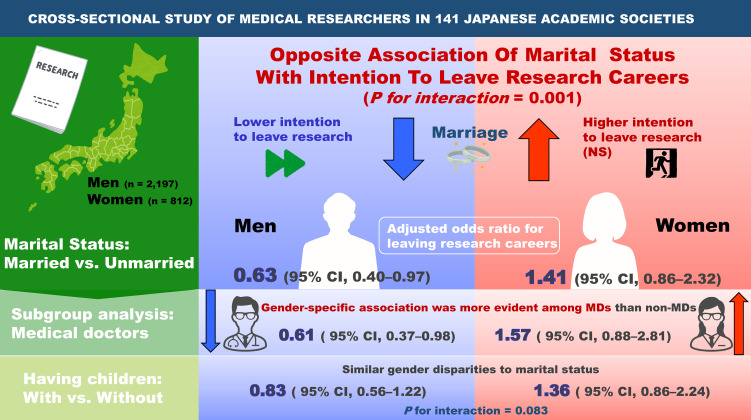

## Background

The decreasing number of medical professionals engaged in research is a global concern [1, 2]. For example, in the United States, the proportion of physicians engaged in research peaked at 4.7% of the total physician workforce in the 1980s and declined to 1.6% by the 2010s [3, 4]. Furthermore, in Japan, overtime work regulations introduced in 2024 are expected to reduce research time [5]. The shortage of medical researchers could lead to a decline in translational and clinical research [6–8]. Therefore, identifying and addressing barriers to research activity is crucial.

Gender disparities in faculty retention rates at medical schools have recently been reported, showing consistently lower retention rates among women faculty compared with men [9]. Given that actual leaving is often preceded by an intention to leave [10], it is important to clarify the factors that lead to gender disparities in intention to leave to develop effective retention strategies in medical fields. Family formation, particularly marriage and childbirth, has been identified as a major cause of career interruptions, especially among women scientists [11, 12]. Several reports on faculty have shown that women spend more time on childcare and domestic labor than men and struggle to balance work and family life [13–15]. Thus, among medical researchers, domestic and childcare responsibilities may have a greater impact on women researchers’ intention to leave their research careers compared with men. However, it remains unclear how family environment uniquely shapes this intention between men and women. If marriage affects the intention to leave research careers, especially among women, the evidence could inform early interventions to retain women researchers before they leave academic medicine.

This study aimed to examine the gender-specific association of marital status and having children with the intention to leave research careers among medical researchers, using a nationwide survey.

## Methods

### Study design

We conducted a secondary analysis of data from a web-based cross-sectional survey administered to medical researchers using a self-administered questionnaire between December 14, 2022 and January 17, 2023. The details of this web-based survey have been previously described [16]. This survey aimed to explore the current state and challenges of medical research and researcher evaluation. The study protocol was approved by the Institutional Review Board of the National Centre for Global Health and Medicine (NCGM-S-004 530–01). In this web-based survey study, respondents were presented with options to consent or decline participation regarding the research content, data collection, and data utilization on the website, and appropriate consent was considered to be obtained when respondents clicked the “agree” button. This approach complies with the Guidance of the Ethical Guidelines for Medical and Biological Research Involving Human Subjects [17].

### Participants

We requested the Japanese Association of Medical Sciences, which consists of 141 medical academic societies (including the Japan Society of Internal Medicine, Japan Surgical Society, and Japanese Society of Public Health), to have each medical society distribute the online questionnaire to its members.

Respondents who did not consent to participate or whose daily work was unrelated to research were excluded. Participants on leave (e.g., maternity or paternity) were instructed to respond based on their work prior to leave. Additionally, individuals who provided the same answer to all questions, indicating possible invalid responses, were excluded. Furthermore, participants who indicated a gender other than men or women, selected “others” or “prefer not to answer” for marital status, or chose “prefer not to answer” regarding having children were excluded.

### Measurements

#### Gender

Gender was assessed by self-report using a binary question in the survey questionnaire. In this study, we use the term “gender” to reflect socially constructed roles and expectations associated with being women or men, rather than biological sex.

#### Marital status and having children

Exposures were marital status and having children. Participants chose one of six marital status options: single, divorced, widowed, married, prefer not to answer, or other. After excluding those who chose “prefer not to answer” or “other,” participants were categorized into two groups based on the presence of a spouse: (1) unmarried (single/divorced/widowed) and (2) married. Participants indicated the number of their children using six options: none, one, two, three, four or more, and prefer not to answer. After excluding those who chose “prefer not to answer,” participants were categorized into two groups: those with and without children.

#### Outcome

The outcome was the intention to leave research careers, measured using one question as described in the previous study [18]. Briefly, participants were asked, “Do you intend to continue your career as a researcher?” and selected one of six options: strongly agree, moderately agree, neither agree nor disagree, moderately disagree, strongly disagree, or uncertain. The intention to stay is significantly and negatively correlated with the intention to leave the workplace among healthcare workers [19]. Therefore, participants were categorized into two groups: those who selected “moderately disagree” or “strongly disagree” were classified as intending to leave research careers. In contrast, the others were classified as having no intention to leave research careers.

#### Mediator

Job satisfaction was considered a potential mediator, based on previous studies suggesting that the positive correlation between work-family conflict and intention to leave the workplace is mediated by decreased job satisfaction [20, 21]. Participants were asked, “How satisfied are you with your current job? (If you have multiple workplaces, please indicate your overall satisfaction level)” and selected one of five options: very satisfied, moderately satisfied, neither satisfied nor dissatisfied, moderately dissatisfied, very dissatisfied. Participants were categorized into two groups: those who selected “moderately dissatisfied” or “very dissatisfied” were classified as dissatisfied with their jobs. In contrast, the others were classified as either satisfied or neutral. The single-item job satisfaction measure is considered a valid method for assessing job satisfaction, as it has been reported to be comparable with multi-item measures [22, 23].

#### Covariates

The following variables, which were reported as factors that promote or hinder research activities, were prepared as covariates [24–28]: age (<40, 40–49, 50–59, ≥60 years, prefer not to answer), research field (clinical medicine, basic medicine, social medicine, others), medical license (medical doctor [MD], non-MD), educational background (undergraduate [4 years], undergraduate [6 years], graduate school [master’s course], graduate school [doctoral course], others), employment status (full-time [tenured], full-time [untenured], part-time, student, others), research effort within the primary affiliated organization (≤10%, approximately 20–30%, approximately 40–60%, ≥70%, prefer not to answer), research difficulties due to insufficient funding in the past year (a lot, a little, no, do not know/not applicable), and academic rank (professor level [e.g., university president, director of research institute, professor, and hospital director], associate professor level [e.g., associate professor, lecturer, and senior researcher at a research institute], assistant professor and post-doc level, others). Affiliation (university, university hospital, other hospital or clinic, public research institution, others) was also evaluated to understand the characteristics of the participants.

### Statistical analysis

Participant characteristics are shown as numbers (%). The gender-specific association of marital status with the intention to leave research careers was evaluated using a multivariate logistic regression model adjusting for all covariates. Subsequently, the analysis was repeated for having children. Both multiplicative and additive interactions [29] of gender on the association between marital status or having children and the intention to leave research careers were assessed. The multiplicative interaction was assessed using interaction terms. The additive interaction was estimated as the relative excess risk due to interaction [30]. Subsequently, analyses were conducted after redefining the exposure as combined categories of marital status and having children to assess how different family compositions affected the outcomes.

Lastly, to explore potential pathways underlying the association between intention to leave and marital status, mediation analysis was conducted as a post-hoc analysis to assess the role of job satisfaction as a mediator. This analysis allowed us to decompose the total association odds ratio (OR) into the direct association OR (the association of marriage with the intention to leave research through pathways not mediated by job satisfaction) and the indirect association OR (the association of marriage with the intention to leave research through pathways mediated by job satisfaction).

For post hoc analyses, the main analyses were repeated in several approaches. First, as a sensitivity analysis, we excluded widowed or divorced participants because they had a high proportion of individuals intending to leave research careers. Second, we excluded participants with very low research effort (≤10%), who may be primarily clinicians rather than researchers. Third, we excluded participants who selected “neither agree nor disagree” for the outcome because neutral response options may represent heterogeneous interpretations and their inclusion can affect dichotomization of Likert-type responses. In addition, we conducted an ordinal logistic regression analysis using all five response categories to preserve the ordinal nature of the outcome. Lastly, we conducted subgroup analyses by medical license status (MD vs. Non-MD) to explore the heterogeneity in the influence of marital status because the interpretation of the intention to leave research careers may differ substantially between these two groups. Two-sided *P* values <0.05 were considered statistically significant. Statistical analyses were performed using Stata BE version 18.0 (StataCorp, College Station, TX, USA), with causal mediation analysis conducted using the “mediate” command.

## Results

### Participant characteristics

Figure S1 shows the flowchart of respondents. Of the 3,555 total respondents, we sequentially excluded 386 respondents who did not consent to participate or whose daily work was unrelated to the study, 30 due to invalid responses, and 43, 64, and 23 based on their answers regarding gender, marital status, and having children, respectively. Consequently, 3,009 participants were included in the final analysis.

Table 1 presents the gender-specific participant characteristics stratified by intention to leave research careers. Men intending to leave research careers had a higher proportion of unmarried individuals and a lower proportion of those with children. On the contrary, among women, the group of participants intending to leave had a lower proportion of unmarried individuals and a higher proportion of those with children. Regarding employment status, women intending to leave had a lower proportion of full-time (tenured) and a higher proportion of part-time employment. In contrast, no such difference was observed among men. Regarding academic rank, men with intention to leave had a lower proportion at the professor level and a higher proportion at the assistant professor and post-doc levels; however, no such difference was observed among women. The characteristics of participants stratified by the intention to leave were similar between men and women across age, research field, medical license, educational background, affiliation, research effort, and insufficient research funding.

**Table 1:** Participant characteristics by the intention to leave research, stratified by gender.

	**Men (n = 2,197)**		**Women (n = 812)**	
	**Intention to ** **leave research** **(n = 247)**	**No intention to ** **leave research** **(n = 1,950)**	**Intention to ** **leave research** **(n = 95)**	**No intention to ** **leave research** **(n = 717)**
**Age, years**				
<40	55 (22.3)	368 (18.9)	34 (35.8)	180 (25.1)
40–49	90 (36.4)	590 (30.3)	24 (25.3)	240 (33.5)
50–59	55 (22.3)	552 (28.3)	25 (26.3)	199 (27.8)
60≤	47 (19.0)	436 (22.4)	12 (12.6)	93 (13.0)
Prefer not to answer	0 (0)	4 (0.2)	0 (0)	5 (0.7)
**Marital status**				
Single	28 (11.3)	148 (7.6)	20 (21.1)	216 (30.1)
Married	212 (85.8)	1,770 (90.8)	63 (66.3)	436 (60.8)
Widowed	2 (0.8)	3 (0.2)	3 (3.2)	9 (1.3)
Divorced	5 (2.0)	29 (1.5)	9 (9.5)	56 (7.8)
**Having children**				
No	46 (18.6)	325 (16.7)	35 (36.8)	336 (46.9)
Yes	201 (81.4)	1,625 (87.3)	60 (63.2)	381 (53.1)
One	40 (19.9)	377 (23.2)	24 (40.0)	137 (36.0)
Two	106 (52.7)	823 (50.7)	26 (43.3)	174 (45.7)
Three	44 (21.9)	356 (21.9)	9 (15.0)	65 (17.1)
More than three	11 (5.5)	69 (4.3)	1 (1.7)	5 (1.3)
**Research field**				
Clinical medicine	185 (74.9)	1,088 (55.8)	67 (70.5)	288 (40.2)
Basic medicine	38 (15.4)	495 (25.4)	9 (9.5)	124 (17.3)
Social medicine	22 (8.9)	310 (15.9)	11 (11.6)	200 (27.9)
Others	2 (0.8)	57 (2.9)	8 (8.4)	105 (14.6)
**Medical license**				
MD	225 (91.1)	1,486 (76.2)	78 (82.1)	365 (50.9)
Non-MD	22 (8.9)	464 (23.8)	17 (17.9)	352 (49.1)
**Educational background**				
Undergraduate (4 years)	4 (1.6)	28 (1.4)	5 (5.3)	36 (5.0)
Undergraduate (6 years)	89 (36.0)	410 (21.0)	35 (36.8)	137 (19.1)
Graduate school (master’s course)	9 (3.6)	100 (5.1)	5 (5.3)	81 (11.3)
Graduate school (doctoral course)	145 (58.7)	1,400 (71.8)	47 (49.5)	447 (62.3)
Others	0 (0.0)	12 (0.6)	3 (3.2)	16 (2.2)
**Affiliation**				
University	63 (25.5)	740 (38.0)	30 (31.6)	328 (45.8)
University hospital	64 (25.9)	442 (22.7)	24 (25.3)	126 (17.6)
Other hospital or clinic	96 (38.9)	565 (29.0)	33 (34.7)	155 (21.6)
Public research institution	7 (2.8)	79 (4.1)	1 (1.1)	45 (6.3)
Others	17 (6.9)	124 (6.4)	7 (7.4)	63 (8.8)
**Employment status**				
Full-time, tenured	162 (65.6)	1,291 (66.2)	48 (50.5)	433 (60.4)
Full-time, untenured	62 (25.1)	530 (27.2)	27 (28.4)	194 (27.1)
Part-time	13 (5.3)	92 (4.7)	14 (14.7)	63 (8.8)
Student	10 (4.1)	22 (1.1)	5 (5.3)	22 (3.1)
Others	0 (0.0)	15 (0.8)	1 (1.1)	5 (0.7)
**Research effort, %**				
≤10	148 (59.9)	632 (32.4)	50 (52.6)	235 (32.8)
20–30	56 (22.7)	653 (33.5)	21 (22.1)	203 (28.3)
40–60	20 (8.1)	428 (22.0)	13 (13.7)	162 (22.6)
70≤	23 (9.3)	234 (12.0)	10 (10.5)	116 (16.2)
Prefer not to answer	0 (0.0)	3 (0.2)	1 (1.1)	1 (0.1)
**Research difficulties due to insufficient research funding**				
A lot	27 (10.9)	288 (14.8)	11 (11.6)	94 (13.1)
A little	51 (20.7)	557 (28.6)	14 (14.7)	171 (23.9)
No	93 (37.7)	797 (40.9)	30 (31.6)	293 (40.9)
Do not know/not applicable	76 (30.8)	308 (15.8)	40 (42.1)	159 (22.2)
**Academic rank**				
Professor level	87 (35.2)	874 (44.8)	23 (24.2)	177 (24.7)
Associate professor level	50 (20.2)	492 (25.2)	18 (19.0)	166 (23.2)
Assistant professor and post-doc level	85 (34.4)	397 (20.4)	35 (36.8)	235 (32.8)
Others	25 (10.1)	187 (9.6)	19 (20.0)	139 (19.4)
**Job satisfaction**				
Very satisfied	27 (10.9)	410 (21.0)	8 (8.4)	126 (17.6)
Moderately satisfied	100 (40.5)	1,092 (56.0)	43 (45.3)	359 (50.1)
Neither satisfied nor dissatisfied	52 (21.1)	289 (14.8)	16 (16.8)	150 (20.9)
Moderately dissatisfied	35 (14.2)	113 (5.8)	19 (20.0)	57 (8.0)
Very dissatisfied	33 (13.4)	46 (2.4)	9 (9.5)	25 (3.5)

Data are n (%). Percentages may not sum to exactly 100% due to rounding.

Abbreviations: MD, medical doctor.

### Association of marital status and having children with the intention to leave research careers

Table 2 shows the gender-specific association of marital status and having children with the intention to leave research careers. The association of marital status with the intention to leave was in opposite direction between men and women with significant gender interactions on both multiplicative (*P* = 0.015) and additive scales (*P* = 0.001). That is, married men showed a significantly lower intention to leave research careers than unmarried men (adjusted odds ratio [aOR] = 0.63, 95% confidence interval [CI]: 0.40–0.97; *P* = 0.038). In contrast, married women exhibited a higher intention to leave research careers than unmarried women, although the difference was not significant (aOR [95% CI], 1.41 [0.86–2.32]; *P* = 0.18).

**Table 2:** Gender-specific adjusted odds ratios of leaving research careers by marital status and having children.

	**Men (n = 2,163)**			**Women (n = 807)**			***P* value for multiplicative interaction**	***P* value for additive interaction**
	**No. of case/No. ** **of participant**	aOR (95% CI)*	***P* value**	**No. of case/No. ** **of participant**	aOR (95% CI)*	***P* value**		
**Marital status**								
Unmarried	35/213	1 (Reference)		32/313	1 (Reference)		0.015	0.001
Married	212/1,950	0.63 (0.40–0.97)	0.038	63/494	1.41 (0.86–2.32)	0.18		
**Having children**								
No	46/364	1 (Reference)		35/371	1 (Reference)		0.13	0.083
Yes	201/1,799	0.83 (0.56–1.22)	0.34	60/436	1.36 (0.83–2.24)	0.22		

Abbreviations: aOR, adjusted odds ratio; CI, confidence interval.

*Adjusted for age, research field, medical license, educational background, employment status, research effort, insufficient research funding, and academic rank.

Similar results to marital status were observed for having children among both genders, although effect modification was not statistically significant (*P* for multiplicative interaction = 0.13; *P* for additive interaction = 0.083). Men with children had a lower intention to leave than those without children (aOR [95% CI], 0.83 [0.56–1.22]; *P* = 0.34). In contrast, women with children had a higher intention to leave than those without children (aOR [95% CI], 1.36 [0.83–2.24]; *P* = 0.22).

In a sensitivity analysis limited to participants who were either single or married, the gender-specific association of marital status and having children with the intention to leave research careers remained robust (Table S1).

After excluding 1,065 participants with very low research effort (≤10%), the point estimates were not largely changed, although interactions by gender became non-significant for marital status (Table S2).

After excluding participants who selected “neither agree nor disagree” to the outcome question on intention to continue research careers (n = 518), the gender-specific association of marital status and having children with the intention to leave research careers remained robust (Table S3). In an ordinal logistic regression analysis using all five response categories of the outcome, the interaction by gender was no longer statistically significant for marital status (Table S4). Point estimates among men were largely unchanged, albeit non-significant. In contrast, the higher point estimates among women were attenuated and became null. This attenuation may be due to the fact that group differences were mainly observed in the highest intention-to-leave categories, while lower and middle categories showed similar distributions (Table S5).

In subgroup analyses by medical license status, the overall proportion of intention to leave was 14.1% among MDs and 4.6% among non-MDs. The gender-specific associations of marital status and having children with the intention to leave research careers remained robust among MD researchers (Table S6). In contrast, among non-MD researchers, the associations were attenuated, especially for marital status, and the interaction by marital status became non-significant (Table S7).

### Combined association of marital status and having children with the intention to leave research careers

Table 3 shows the gender-specific combined association of marital status and having children with the intention to leave research careers. Compared with unmarried men without children, married men showed a lower intention to leave research careers, regardless of whether they had children or not (aOR [95% CI]; 0.63 [0.39–1.04] and 0.58 [0.29–1.16], respectively). In contrast, compared with unmarried women without children, those with all other combinations of marital status and having children tended to be associated with a higher intention to leave research careers.

**Table 3:** Gender-specific adjusted odds ratios of leaving research by combinations of marital status and having children.

	**Men (n = 2,163)**			**Women (n = 807)**		
	**No. of case/No. ** **of participant**	aOR (95% CI)*	***P* value**	**No. of case/No. ** **of participant**	aOR (95% CI)*	***P* value**
Unmarried without children	29/178	1 (Reference)		22/261	1 (Reference)	
Married without children	17/186	0.58 (0.29–1.16)	0.12	13/110	2.10 (0.95–4.68)	0.068
Unmarried with children	6/35	1.01 (0.35–2.89)	0.98	10/52	2.28 (0.91–5.72)	0.080
Married with children	195/1,764	0.63 (0.39–1.04)	0.071	50/384	1.66 (0.92–3.00)	0.092

Abbreviations: aOR, adjusted odds ratio; CI, confidence interval.

*Adjusted for age, research field, medical license, educational background, employment status, research effort, insufficient research funding, and academic rank.

In a sensitivity analysis limited to participants who were either single or married, the gender-specific combined association of marital status and having children with the intention to leave research careers were materially unchanged (Table S8).

### Mediation analysis of job satisfaction

Table 4 shows the results of the median analysis for the pathway between marital status and the intention to leave research careers. For both men and women, job satisfaction suggested a mediating role in the association between marriage and intention to leave. The indirect associations of marriage with intention to leave through job satisfaction were significantly negative (aOR [95% CI]; 0.89 [0.81–0.98] and 0.90 [0.81–1.00], respectively). Furthermore, although not statistically significant, marriage was linked to decreased intention to leave irrespectively of job satisfaction among men; the direct association tended to be negative (aOR [95% CI], 0.75 [0.52–1.10]; *P* = 0.15). Conversely, in women, marriage tended to be linked to increased intention to leave irrespectively of job satisfaction; the direct association tended to be positive (aOR [95% CI], 1.52 [0.96–2.39]; *P* = 0.072). In a sensitivity analysis limited to participants who were either single or married, the results of the median analysis remained robust (Table S9).

**Table 4:** Mediating role of job satisfaction between marital status and the intention to leave research careers.

	**Total association**		**Natural direct association**		**Natural indirect association**	
	aOR (95% CI)*	***P* value**	aOR (95% CI)*	***P* value**	aOR (95% CI)*	***P* value**
Men (n = 2,163)						
Unmarried	1.00		1.00		1.00	
Married	0.67 (0.45–0.99)	0.042	0.75 (0.52–1.10)	0.15	0.89 (0.81–0.98)	0.013

Women (n = 805)						
Unmarried	1.00		1.00		1.00	
Married	1.37 (0.87–2.14)	0.18	1.52 (0.96–2.39)	0.072	0.90 (0.81–1.00)	0.046

Abbreviations: aOR, adjusted odds ratio; CI, confidence interval.

*Adjusted for age, research field, medical license, educational background, employment status, research effort, insufficient research funding, and academic rank.

## Discussion

To our knowledge, this is the first study to examine the gender-specific association of marital status and having children with the intention to leave research careers among medical researchers. Our findings of the significant gender interactions suggest that marital status is linked to the intention to leave research careers in opposite directions for men and women. Compared with unmarried men, married men were less likely to have the intention to leave research careers. In contrast, married women showed a higher point estimate for the intention to leave compared with unmarried women, although this difference was not significant. Mediation analysis suggested that, for both genders, job satisfaction was associated with a lower intention to leave in relation to marriage. However, in women, other pathways contributed to a greater intention to leave, resulting in different total associations by gender.

Our findings indicate that the association between marriage and the intention to leave research careers was in opposite directions for men and women. One possible explanation for this gender difference is that marriage may coincide with additional social and professional burdens for women. Although work-life imbalance or work-family conflict was not directly measured in this study, as with women in the broader workforce [31], prior studies among physicians [32, 33] or researchers [34, 35] suggest that women often bear a disproportionate share of domestic responsibilities, which may constrain the time available for research and career advancement. Structural barriers in academia, including fixed-term contracts, limited promotion opportunities, and geographic constraints related to coordination of dual-earner careers [34, 36–38] may further interact with life-course transitions such as marriage and parenthood for women. In contrast, the finding among men is consistent with prior studies suggesting that marriage does not appear to disadvantage men and may even be associated with greater career stability or a higher likelihood of achieving tenure [39, 40]. While these mechanisms were not directly assessed, they may contribute to the observed gender differences in intention to leave.

The gender-specific association between marriage and intention to leave research careers was primarily observed among MD researchers, although caution should be exercised in interpreting the results of non-MD researchers as the number of cases in each category was very small. Nonetheless, these results may reflect the professional structure of medicine. Physicians may achieve greater lifetime earnings through clinical practice than through research-oriented career paths [41]. Because MDs can rely on clinical practice as an alternative career pathway, they may be less likely to perceive leaving research as a financial risk compared with non-MD researchers. Indeed, majority of physician scientists in Japan return to clinical practice after leaving research [42]. In the context of recent work-style reforms limiting working hours in Japan, leaving research may become an increasingly attractive career option. In contrast, non-MD researchers may have fewer alternative professional pathways outside academia than MDs and may face greater career and employment instability [43, 44]. These points might be supported by the fact that intention to leave was generally lower among non-MDs than MDs in our study. Additional large-scale studies are warranted to confirm the medical license specific associations.

The present study also highlights the potential role of job satisfaction in shaping intention to leave research careers. In both men and women, marriage was indirectly associated with a lower intention to leave through higher job satisfaction. This is consistent with previous studies showing that lower job satisfaction is associated with higher intention to leave among healthcare professionals and university faculty [45, 46]. However, the total associations differed by gender. Among men, the protective indirect association through satisfaction was consistent with the overall lower intention to leave associated with marriage. Among women, although job satisfaction was similarly protectively associated with a lower intention to leave, it did not fully offset the higher overall intention to leave due to other factors. These findings suggest that job satisfaction represents a potentially modifiable factor for retention, although broader structural conditions may also need to be addressed.

Our results regarding the combined associations of marital status and having children further support this interpretation. Among men, compared with unmarried researchers without children, married researchers were less likely to intend to leave research regardless of whether they had children. In contrast, among women, compared with unmarried women researchers without children, women researchers in all other categories with a spouse or children tended to be more likely to express an intention to leave. These findings differ from previous studies conducted mainly in Western countries, which have suggested that having children, rather than marriage, contributes to gender disparities in time spent on work [33] and in obtaining tenure-track positions [11]. Our findings suggest that the burdens associated with marriage may already be substantial enough to increase the intention to leave research careers, such that having children does not significantly alter this trend. This interpretation is consistent with the persistent gender imbalance in domestic labor in Japan [47, 48] and could help explain why marriage tends to be associated with a higher intention to leave research careers, regardless of having children.

In our study, most sensitivity analyses supported the main results. However, in the ordinal logistic regression analysis treating the outcome as an ordinal variable, the associations among women became null. This may be because ordinal logistic regression primarily captures monotonic shifts across outcome categories; in the present study, the proportion of lower and middle categories of the outcome was similar between married and unmarried women, whereas differences were mainly observed in the highest categories.

A strength of this study is its comprehensive investigation of diverse medical researchers, targeting over 100 medical academic societies through the Japanese Association of Medical Sciences, unlike previous studies that focused solely on medical faculty or MDs. This study has several limitations. First, because this was a cross-sectional study, we cannot establish a causal relationship between marital status and intention to leave research careers. Reverse causation is particularly plausible among men. Specifically, men researchers in stable positions with a strong intention to continue their research careers may be more likely to marry due to their stability. Therefore, the lower intention to leave research observed among married men may reflect such selection bias rather than a causal protective effect of marriage. Furthermore, the temporal sequence between job satisfaction and the intention to leave could not be determined, and the mediation analysis involving job satisfaction was exploratory. Second, since work–life balance and related factors, as well as qualitative data on the reasons for participants’ intention to leave, were not measured, the mechanisms underlying the observed gender differences could not be fully assessed. Further studies are needed to clarify the factors contributing to these differences. Third, the participants in this study represent only a portion of Japanese medical researchers, and individuals with negative attitudes toward research have been less likely to respond to the questionnaire survey. However, the participant demographics, such as the higher proportion of women who were single and without children compared with men, were consistent with previous reports on the careers of scientists [11, 49] and physicians [33, 50]. Forth, the outcome in this study was the intention to leave research careers rather than actual leaving. Although intention to leave among healthcare professionals has been associated with later actual leaving [10], these two constructs are not identical, and further research is needed to determine whether intention translates into actual resignation. Fifth, this study may be subject to survivorship bias. Because the study targets researchers currently affiliated with academic societies, those who have already left research due to marriage or childcare are not included. This selection bias may lead to an underestimation of the gender disparity. Sixth, since this study focused on Japanese medical researchers, the findings may not be generalizable to other countries with different gender roles and public policies. Seventh, while we adjusted for several known confounding factors, unmeasured confounders, such as social support, may not have been assessed in the questionnaire survey.

## Conclusions

This study examined the gender-specific association between marital status and having children with the intention to leave research careers among medical researchers. The significant interaction by gender indicates that the impact of marriage on the intention to leave research careers differs between men and women, with this pattern primarily observed among MD researchers. This study suggests the importance of considering gender difference when developing research career retention strategies for medical researchers.

## Abbreviations

CI: confidence interval
MD: medical doctor
OR: odds ratio

## Supplementary information

**Additional file 1:** **Table S1.** Gender-specific adjusted odds ratios for the intention to leave research careers, stratified by marital and parental status, excluding widowed and divorced participants. **Table S2.** Gender-specific adjusted odds ratios of leaving research careers by marital status and having children, excluding participants with ≤10% research effort. **Table S3.** Gender-specific adjusted odds ratios of leaving research careers by marital status and having children, excluding participants with neither intention. **Table S4.** Gender-specific adjusted odds ratios of leaving research careers by marital status and having children in an ordinal logistic regression. **Table S5.** Distribution of intention to continue research by gender and marital status. **Table S6.** Gender-specific adjusted odds ratios of leaving research careers by marital status and having children among MD researchers. **Table S7.** Gender-specific adjusted odds ratios of leaving research careers by marital status and having children among non-MD researchers. **Table S8.** Gender-specific adjusted odds ratios for the intention to leave research careers, stratified by combinations of marital status and having children, excluding widowed and divorced participants. **Table S9.** Association of job satisfaction as a mediator between marital status and the intention to leave research careers, excluding widowed and divorced participants. **Figure S1.** Flowchart illustrating participant recruitment.

## Declarations

### Availability of data and material

Data in this study will be made available to researchers upon reasonable request, subject to ethical guidelines and approval from the ethics committee.
