# Supplementary material for: Gender disparities in the association between marital status and intention to leave research careers among medical researchers
Source: Environ Health Prev Med. 2026 May 9;31:30. doi: 10.1265/ehpm.25-00407 (PMC13171253; doi:10.1265/ehpm.25-00407)
Supplement: Supplementary file 1 — Additional file 1: Table S1. Gender-specific adjusted odds ratios for the intention to leave research careers, stratified by marital and parental status, excluding widowed and divorced participants. Table S2. Gender-specific adjusted odds ratios of leaving research careers by marital status and having children, excluding participants with ≤10% research effort. Table S3. Gender-specific adjusted odds ratios of leaving research careers by marital status and having children, excluding participants with neither intention. Table S4. Gender-specific adjusted odds ratios of leaving research careers by marital status and having children in an ordinal logistic regression. Table S5. Distribution of intention to continue research by gender and marital status. Table S6. Gender-specific adjusted odds ratios of leaving research careers by marital status and having children among MD researchers. Table S7. Gender-specific adjusted odds ratios of leaving research careers by marital status and having children among non-MD researchers. Table S8. Gender-specific adjusted odds ratios for the intention to leave research careers, stratified by combinations of marital status and having children, excluding widowed and divorced participants. Table S9. Association of job satisfaction as a mediator between marital status and the intention to leave research careers, excluding widowed and divorced participants. Figure S1. Flowchart illustrating participant recruitment. [file ehpm-31-030-s001.docx]

**Table S1.** Gender-specific adjusted odds ratios for the intention to leave research careers, stratified by marital and parental status, excluding widowed and divorced participants.

|  | Men (n = 2,124) | | | Women (n = 730) | | | *P* value for multiplicative interaction | *P* value for additive interaction |
| --- | --- | --- | --- | --- | --- | --- | --- | --- |
|  | No. of case/No. of participant | aOR (95% CI)^*^ | *P* value | No. of case/No. of participant | aOR (95% CI)^*^ | *P* value |  |  |
| **Marital status** |  |  |  |  |  |  |  |  |
| Single | 28/174 | 1 (Reference) |  | 20/236 | 1 (Reference) |  | 0.010 | <0.001 |
| Married | 212/1,950 | 0.64 (0.39-1.05) | 0.078 | 63/494 | 1.74 (0.95-3.18) | 0.073 |  |  |
| **Having children** |  |  |  |  |  |  |  |  |
| No | 45/356 | 1 (Reference) |  | 32/342 | 1 (Reference) |  | 0.15 | 0.11 |
| Yes | 195/1,768 | 0.82 (0.55-1.21) | 0.31 | 51/388 | 1.36 (0.79-2.32) | 0.27 |  |  |
| Abbreviations: aOR, adjusted odds ratio; CI, confidence interval.  ^*^Adjusted for age, research field, medical license, educational background, employment status, research effort, insufficient research funding, and academic rank. | | | | | | | | |

**Table S2**. Gender-specific adjusted odds ratios of leaving research careers by marital status and having children, excluding participants with ≤10% research effort.

|  | Men (n = 1,396) | | | Women (n = 524) | | | *P* value for multiplicative interaction | *P* value for additive interaction |
| --- | --- | --- | --- | --- | --- | --- | --- | --- |
|  | No. of case/No. of participant | aOR (95% CI)^*^ | *P* value | No. of case/No. of participant | aOR (95% CI)^*^ | *P* value |  |  |
| **Marital status** |  |  |  |  |  |  |  |  |
| Unmarried | 16/152 | 1 (Reference) |  | 17/208 | 1 (Reference) |  | 0.23 | 0.15 |
| Married | 83/1,244 | 0.80 (0.43-1.50) | 0.49 | 28/316 | 1.38 (0.66-2.90) | 0.39 |  |  |
| **Having children** |  |  |  |  |  |  |  |  |
| No | 23/258 | 1 (Reference) |  | 18/259 | 1 (Reference) |  | 0.12 | 0.076 |
| Yes | 76/1,138 | 0.83 (0.48-1.44) | 0.52 | 27/265 | 1.70 (0.81-3.58) | 0.16 |  |  |
| Abbreviations: aOR, adjusted odds ratio; CI, confidence interval.  ^*^Adjusted for age, research field, medical license, educational background, employment status, research effort, insufficient research funding, and academic rank. | | | | | | | | |

**Table S3**. Gender-specific adjusted odds ratios of leaving research careers by marital status and having children, excluding participants with neither intention.

|  | Men (n = 1,803) | | | Women (n = 655) | | | *P* value for multiplicative interaction | *P* value for additive interaction |
| --- | --- | --- | --- | --- | --- | --- | --- | --- |
|  | No. of case/No. of participant | aOR (95% CI)^*^ | *P* value | No. of case/No. of participant | aOR (95% CI)^*^ | *P* value |  |  |
| **Marital status** |  |  |  |  |  |  |  |  |
| Unmarried | 35/187 | 1 (Reference) |  | 32/246 | 1 (Reference) |  | 0.025 | 0.004 |
| Married | 212/1,616 | 0.63 (0.39-1.00) | 0.051 | 63/409 | 1.50 (0.89-2.55) | 0.13 |  |  |
| **Having children** |  |  |  |  |  |  |  |  |
| No | 46/305 | 1 (Reference) |  | 35/298 | 1 (Reference) |  | 0.15 | 0.10 |
| Yes | 201/1,498 | 0.76 (0.51-1.15) | 0.20 | 60/357 | 1.31 (0.77-2.22) | 0.31 |  |  |
| Abbreviations: aOR, adjusted odds ratio; CI, confidence interval.  ^*^Adjusted for age, research field, medical license, educational background, employment status, research effort, insufficient research funding, and academic rank. | | | | | | | | |

**Table S4**. Gender-specific adjusted odds ratios of leaving research careers by marital status and having children in an ordinal logistic regression.

|  | Men (n = 2,152) | | Women (n = 776) | | *P* value for multiplicative interaction |
| --- | --- | --- | --- | --- | --- |
|  | aOR (95% CI)^*^ | *P* value | aOR (95% CI)^*^ | *P* value |  |
| **Marital status** |  |  |  |  |  |
| Unmarried | 1 (Reference) |  | 1 (Reference) |  | 0.24 |
| Married | 0.80 (0.60-1.06) | 0.12 | 1.00 (0.76-1.32) | 0.99 |  |
| **Having children** |  |  |  |  |  |
| No | 1 (Reference) |  | 1 (Reference) |  | 0.51 |
| Yes | 0.83 (0.67-1.05) | 0.12 | 1.01 (0.77-1.33) | 0.96 |  |
| Abbreviations: aOR, adjusted odds ratio; CI, confidence interval.  ^*^Adjusted for age, research field, medical license, educational background, employment status, research effort, insufficient research funding, and academic rank. | | | | | |

**Table S5.** Distribution of intention to continue research by gender and marital status.

|  | Men (n = 2,152) | | Women (n = 776) | |  |
| --- | --- | --- | --- | --- | --- |
| Do you intend to continue your career as a researcher? | Unmarried (n = 213) | Married (n = 1,939) | Unmarried (n = 296) | Married (n = 480) | |
| Strongly agree | 69 (32.4) | 694 (35.8) | 91 (30.7) | 149 (31.0) | |
| Moderately agree | 82 (38.5) | 695 (35.8) | 106 (35.8) | 182 (37.9) | |
| Neither agree nor disagree | 27 (12.7) | 338 (17.4) | 67 (22.6) | 86 (17.9) | |
| Moderately disagree | 18 (8.5) | 145 (7.5) | 18 (6.1) | 48 (10.0) | |
| Strongly disagree | 17 (8.0) | 67 (3.5) | 14 (4.7) | 15 (3.1) | |
| Data are n (%). Percentages may not sum to exactly 100% due to rounding. | | | | | |

**Table S6**. Gender-specific adjusted odds ratios of leaving research careers by marital status and having children among MD researchers.

|  | Men (n = 1,681) | | | Women (n = 436) | | | *P* value for multiplicative interaction | *P* value for additive interaction |
| --- | --- | --- | --- | --- | --- | --- | --- | --- |
|  | No. of case/No. of participant | aOR (95% CI)^*^ | *P* value | No. of case/No. of participant | aOR (95% CI)^*^ | *P* value |  |  |
| **Marital status** |  |  |  |  |  |  |  |  |
| Unmarried | 30/126 | 1 (Reference) |  | 24/165 | 1 (Reference) |  | 0.010 | <0.001 |
| Married | 195/1,555 | 0.61 (0.37-0.98) | 0.043 | 54/271 | 1.57 (0.88-2.81) | 0.13 |  |  |
| **Having children** |  |  |  |  |  |  |  |  |
| No | 39/229 | 1 (Reference) |  | 26/170 | 1 (Reference) |  | 0.21 | 0.16 |
| Yes | 186/1,452 | 0.85 (0.56-1.30) | 0.46 | 52/266 | 1.35 (0.76-2.39) | 0.31 |  |  |
| Abbreviations: aOR, adjusted odds ratio; CI, confidence interval; MD, medical doctor.  ^*^Adjusted for age, research field, medical license, educational background, employment status, research effort, insufficient research funding, and academic rank. | | | | | | | | |

**Table S7**. Gender-specific adjusted odds ratios of leaving research careers by marital status and having children among non-MD researchers.

|  | Men (n = 460) | | | Women (n = 358) | | | *P* value for multiplicative interaction | *P* value for additive interaction |
| --- | --- | --- | --- | --- | --- | --- | --- | --- |
|  | No. of case/No. of participant | aOR (95% CI)^*^ | *P* value | No. of case/No. of participant | aOR (95% CI)^*^ | *P* value |  |  |
| **Marital status** |  |  |  |  |  |  |  |  |
| Unmarried | 5/83 | 1 (Reference) |  | 8/142 | 1 (Reference) |  | 0.66 | 0.53 |
| Married | 17/377 | 0.89 (0.28-2.86) | 0.85 | 9/216 | 1.09 (0.33-3.61) | 0.89 |  |  |
| **Having children** |  |  |  |  |  |  |  |  |
| No | 7/132 | 1 (Reference) |  | 9/194 | 1 (Reference) |  | 0.63 | 0.52 |
| Yes | 15/328 | 0.83 (0.28-2.47) | 0.74 | 8/164 | 1.26 (0.38-4.19) | 0.71 |  |  |
| Abbreviations: aOR, adjusted odds ratio; CI, confidence interval; MD, medical doctor.  ^*^Adjusted for age, research field, medical license, educational background, employment status, research effort, insufficient research funding, and academic rank. | | | | | | | | |

**Table S8.** Gender-specific adjusted odds ratios for the intention to leave research careers, stratified by combinations of marital status and having children, excluding widowed and divorced participants.

|  | Men (n = 2,120) | | | Women (n = 730) | | |
| --- | --- | --- | --- | --- | --- | --- |
|  | No. of case/No. of participant | aOR (95% CI) ^*^ | *P* value | No. of case/No. of participant | aOR (95% CI) ^*^ | *P* value |
| Single without children | 28/170 | 1 (Reference) |  | 19/232 | 1 (Reference) |  |
| Married without children | 17/186 | 0.57 (0.29-1.15) | 0.12 | 13/110 | 2.21 (0.95-5.09) | 0.064 |
| Single with children | 0/4 | NA | - | 1/4 | 8.90 (0.80-99.44) | 0.076 |
| Married with children | 195/1,764 | 0.63 (0.39-1.04) | 0.069 | 50/384 | 1.77 (0.93-3.35) | 0.080 |
| Abbreviations: aOR, adjusted odds ratio; CI, confidence interval.  ^*^Adjusted for age, research field, medical license, educational background, employment status, research effort, insufficient research funding, and academic rank. | | | | | | |

**Table S9.** Association of job satisfaction as a mediator between marital status and the intention to leave research careers, excluding widowed and divorced participants.

|  | Total association | | Natural direct association | | Natural indirect association | |
| --- | --- | --- | --- | --- | --- | --- |
|  | aOR (95% CI)^*^ | *P* value | aOR (95% CI)^*^ | *P* value | aOR (95% CI)^*^ | *P* value |
| Men (n = 2,124) |  |  |  |  |  |  |
| Single | 1.00 |  | 1.00 |  | 1.00 |  |
| Married | 0.70 (0.45-1.09) | 0.11 | 0.78 (0.50-1.20) | 0.26 | 0.90 (0.82-0.99) | 0.030 |
|  |  |  |  |  |  |  |
| Women (n = 728) |  |  |  |  |  |  |
| Single | 1.00 |  | 1.00 |  | 1.00 |  |
| Married | 1.68 (0.95-2.96) | 0.073 | 1.90 (1.07-3.37) | 0.028 | 0.88 (0.78-1.00) | 0.043 |
| Abbreviations: aOR, adjusted odds ratio; CI, confidence interval.  ^*^Adjusted for age, research field, medical license, educational background, employment status, research effort, insufficient research funding, and academic rank. | | | | | | |


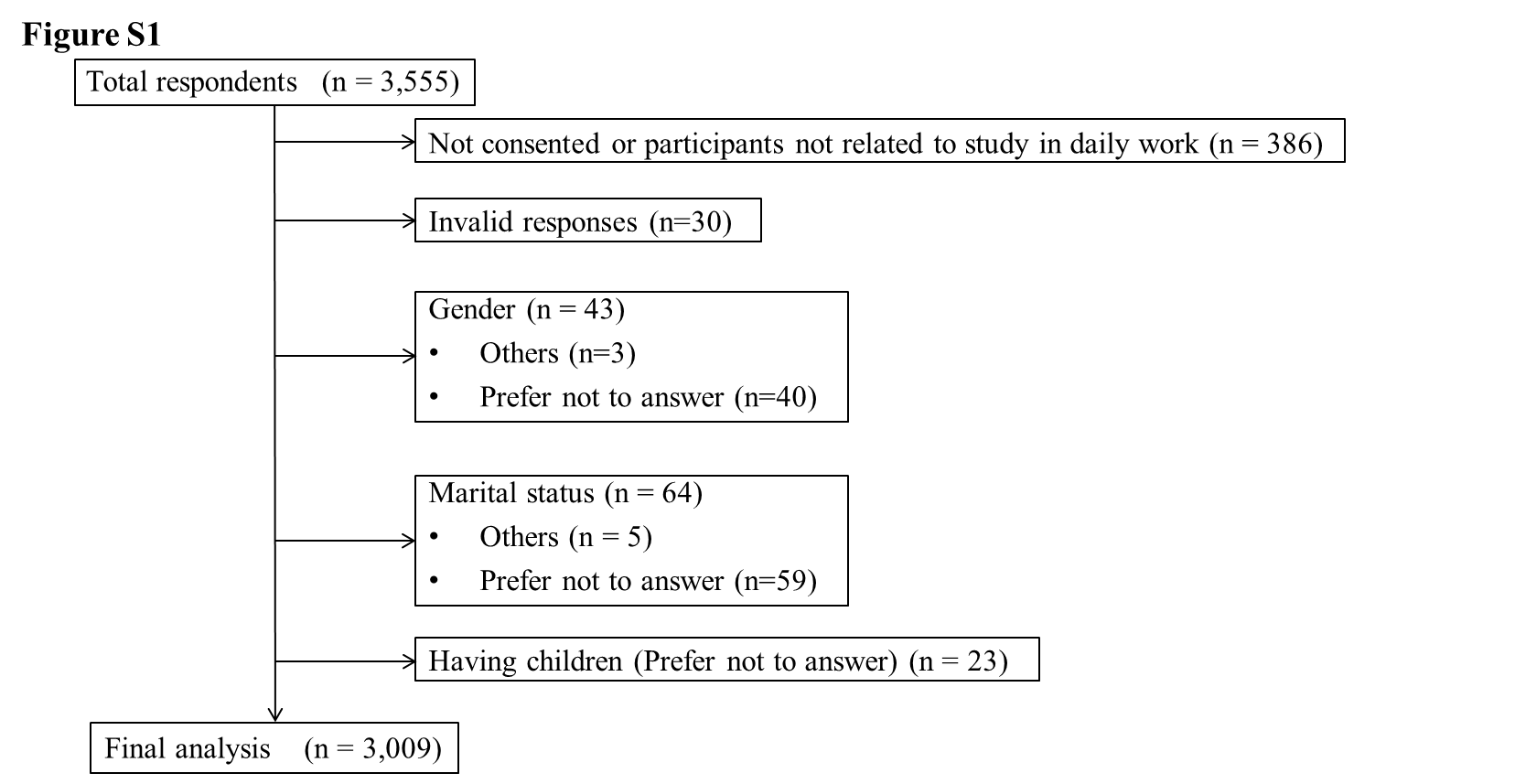


**Figure S1.** Flowchart illustrating participant recruitment
